# Harnessing synergistic potential of plant growth promoting bacteria to mitigate climate-induced stress in plants

**DOI:** 10.3389/fpls.2026.1861876

**Published:** 2026-07-01

**Authors:** Awmpuizeli Fanai, Felicia Lalremruati, Nancy Lalhriatpuii, Lalrokimi, Lalenpuii Zorem, Rosie Lalmuanpuii, Beirachhitha Bohia, Prashant Kumar Singh, Lalrinmawia, Zothanpuia

**Affiliations:** 1 Department of Biotechnology, Mizoram University, Aizawl, Mizoram, India; 2 Department of Biotechnology/Life Sciences, Pachhunga University College (A Constituent College of Mizoram University), Aizawl, Mizoram, India; 3 Department of Botany, Mizoram University, Aizawl, Mizoram, India; 4 Deparment of Zoology, Govt. Zirtiri Residential Science College, Aizawl, Mizoram, India

**Keywords:** bioformulation, climate change, consortia, plant growth-promoting bacteria, stress, synergistic communities

## Abstract

Climate change has emerged as the most pressing global challenge, driving extreme weather events and multifactorial stress combinations (MFSCs) that threaten ecological balance and agricultural sustainability. Plants possess intrinsic defense mechanisms, yet the increasing intensity and frequency of climate-induced stresses often exceed their adaptive capacity. Plant growth-promoting bacteria (PGPB) offer a sustainable solution, as they produce bioactive metabolites that enhance plant resilience to both biotic and abiotic stresses. Importantly, microbial consortia, synergistic communities of PGPB, demonstrate greater effectiveness than single strains, owing to their collective metabolic potential and adaptability across diverse soils and climates. Evidence from microbes isolated from extreme habitats, such as Antarctic plants, and formulations involving *Pseudomonas*, *Bacillus*, *Trichoderma*, and other genera, highlights their capacity to mitigate cold, drought, heat, salinity, and osmotic stresses while promoting plant growth and productivity. These consortia function through mechanisms including nutrient transport, antioxidant activity, systemic resistance, and metabolite accumulation. Thus, deeper molecular-level investigations into PGPB consortia are crucial for developing targeted bioformulations that safeguard agriculture against the impacts of climate change. This review highlights the potential of microbial consortia as sustainable agents for mitigating stress and promoting plant growth under changing global conditions.

## 1 Introduction

Climate change has emerged as the most urgent global issue, which contributes to extreme weather conditions such as increasing temperatures, droughts, heatwaves, tropical typhoons, and changes in rainfall and humidity patterns. It also exacerbates the emergence and spreads of plant pathogens, a process intensified by human activities. This creates a complex environmental stressor known as multifactorial stress combinations (MFSCs). These severe and unpredictable conditions, with their destructive effects, disturb the ecological cycle, where the growing frequency and variety of these stresses may surpass the capacity of living organisms to adapt (Walsh et al., 2020; Bastos et al., 2023).

The agricultural sector is the most vulnerable to significant climate change, which disrupts plant systems. For example, elevated temperatures can increase transpiration rates, leading to water stress. Additionally, heatwaves and droughts can cause physiological stresses that affect photosynthesis in plants. As a result, the farming cycle is disrupted, and the overall quality of crop production decreases. Stress conditions often lead to tissue dehydration, as seen in heat, salt, and drought stress. In response to stress, plants have developed intrinsic defense mechanisms, such as the production of reactive oxygen species and plant hormones, and the use of transcription factors that help them tolerate certain levels of climate-induced stress. However, the high intensity and frequent occurrence of global climate change at the current rate pose significant concerns for plants and other life forms (De Ollas et al., 2023).

Human-driven climate change has an impact on all four pillars of food security, such as availability, access, utilization, and stability, by depressing crop productivity, increasing price volatility, and amplifying undernutrition in low-income populations (Molotoks et al., 2020). Conventional methods such as traditional breeding, irrigation, chemical fertilizers, and basic agronomy remain fundamental in many regions, but they are often slow and ineffective. Although conventional breeding sometimes produces lasting improvements, it is a slow, trial-and-error process because many important stress-tolerance traits are quantitative, heavily influenced by genotype and environment, and therefore have low heritability. These conventional methods typically target a single stress, whereas plants in the field are increasingly exposed to multiple or sequential stresses; therefore, focusing on a single stress tolerance may fail or even worsen outcomes under combined stresses. Chemical fertilizers pose environmental risk, including soil degradation, salinization, pollution, heavy metal accumulation, disruption of soil microbiota, and reduced biodiversity, which weaken plant resilience to drought, heat, and salinity. Climate change worsens these effects by increasing fertilizer inefficiency through volatilization and runoff under variable rainfall and high-temperature conditions. Genetic modification and gene editing are alternative approaches to improve stress resistance. However, due to the complexity and variability of stress traits, as well as high costs, long timelines, potential off-target effects, and crop-specific challenges, they are not yet reliable enough to fully address climate-related stresses. Therefore, there is a growing emphasis on developing biofertilizers, bio-stimulants, biochar, and nano-fertilizers, since conventional fertilizers alone cannot sustainably mitigate stress or support climate-smart productivity (Khan et al., 2025; Guzmán et al., 2021; Ray et al., 2025; Ruffatto et al., 2025).

Beneficial microorganisms like plant growth-promoting microorganism (PGPM) are promising agents that naturally produce bioactive secondary metabolites, aiding plants in adapting to various climate-induced biotic and abiotic stresses through both direct and indirect mechanisms. Additionally, these beneficial bacteria, as a community, have synergistic potential that enhances the production of secondary metabolites, ultimately providing greater benefits to plants compared to using beneficial bacteria as single strains (Fanai et al., 2024). Microbial consortia of plant growth-promoting bacteria are increasingly recognized as sustainable agents that enhance plant growth and mitigate stress under changing environmental conditions. These consortia work synergistically through mechanisms such as improved nutrient transport, antioxidant activity, systemic resistance, and metabolic accumulation, offering advantages over single-strain inoculants (Zhang et al., 2025). Studies showed that Plant growth promoting bacteria (PGPB) consortia can effectively alleviate abiotic stress like drought and combined pollution such as microplastics and heavy metals, thereby improving plant biomass, nutrient uptake, and stability of the soil microbial community (Petrillo et al., 2022; Negi et al., 2024; Fernández et al., 2022). The use of multi-strain consortia has been linked to enhanced seed germination, root development, and stress tolerance in various crops, supporting their role in climate-resilient agriculture. Research also highlights the importance of tailored microbial formulations and delivery methods to maximize efficacy in field conditions. Overall, microbial consortia represent a promising approach for sustainable agriculture by promoting plant growth and protecting crops against diverse stresses exacerbated by climate change (Santoyo et al., 2021).

## 2 Climate-induced stresses in plants

### 2.1 Drought stress

Drought stress severely affects plant systems by causing water scarcity that disrupts morphological, physiological, biochemical, and molecular functions. It induces osmotic imbalance and the accumulation of reactive oxygen species, which damage cellular structures, reduce photosynthesis, and impair the electron transport system (Qiao et al., 2024). Plants respond by altering stomatal conductance to reduce transpiration, modifying root-to-shoot ratios by increasing root length, and accumulating compatible solutes to maintain osmotic balance (Seleiman et al., 2021). The hormone abscisic acid plays a central role in drought response by regulating stomatal closure, root growth, gene expression, and metabolic adjustments to enhance tolerance (Ali et al., 2025). Additionally, drought triggers activation of antioxidant enzymes to mitigate oxidative damage and involves complex signaling pathways, including calcium and Reactive oxygen species (ROS) as secondary messengers (Wahab et al., 2022; Ahluwalia et al., 2021).

### 2.2 Heat stress

Heat stress mainly disrupts plant systems by damaging photosynthesis, protein stability, and membrane integrity. It hampers carbon dioxide assimilation and photochemical reactions by affecting chloroplast components and heat-sensitive proteins like RuBisCo activase, leading to reduced photosynthetic efficiency and potential cell death (Zahra et al., 2022). Heat also causes protein denaturation and membrane disruption, destabilizing cellular structures and accelerating the degradation of chlorophyll and leaf senescence (Kumar et al., 2025). During the reproductive stage, heat stress decreases pollen viability and fertilization success, severely impacting crop yield (Jagadish et al., 2021). Plants respond by activating heat shock proteins (HSPs) that help refold damaged proteins and maintain cellular homeostasis, along with hormonal changes such as increased abscisic acid promoting stomatal closure to reduce water loss. Despite these adaptive mechanisms, heat stress remains a threat to plant growth and productivity (Mondal et al., 2023; Kan et al., 2023).

### 2.3 Salinity

Salinity in plants primarily disrupts growth and development by causing ionic imbalance, osmotic stress, and oxidative damage, which impair physiological processes like seed germination, photosynthesis, and nutrient uptake. Excessive salt ions such as Na+ and Cl- interfere with cellular functions, leading to reduced leaf development, stomatal closure, and lower photosynthetic rates, ultimately threatening crop productivity and food security (Hameed et al., 2021). Plants respond through various mechanisms, including ion homeostasis regulation, osmolyte accumulation, antioxidant defense activation, and modulation of phytohormones that regulate stress response (Joshi et al., 2022). Salinity also affects chloroplast structure and function by altering size, number, membrane organization, and biochemical reactions essential for photosynthesis; halophytes show distinct adaptations compared to glycophytes.

### 2.4 Waterlogging and flooding

Hypoxic or anoxic conditions are the most severe impacts of flooding on plants. They limit oxygen availability to the roots, disrupting aerobic respiration and energy metabolism. This affects all growth stages from seed germination to maturity, leading to reduced photosynthesis, altered hormone signaling, notably ethylene, gibberellins, and abscisic acid, and changes in root architecture such as adventitious root and aerenchyma formation to improve oxygen diffusion (Mudasir and Shahzad, 2025; Manghwar et al., 2024). Waterlogged soils also cause nutrient imbalances and nitrogen losses through processes like denitrification, further impairing plant development and crop yields (Kaur et al., 2020). Plants respond by shifting metabolism towards anaerobic respiration and activating specific gene networks involved in stress perception and adaptation, including transcription factors related to ethylene response and carbohydrate metabolism (Gui et al., 2024). Despite advances in molecular understanding, further research is needed on gene regulatory networks and practical applications of multi-omics approaches to enhance waterlogging tolerance in plants (Mudasir and Shahzad, 2025).

### 2.5 Cold and chilling stress

Cold and chilling stress significantly limit plant growth, development, and crop yield by disrupting physiological and molecular processes. Plants respond through cold acclimation involving membrane modifications, cytoskeletal rearrangement, and activation of calcium signaling pathways that trigger downstream protein kinases and transcription factors such as ICE1-CBF-COR to regulate gene expression for cold tolerance (Zhou et al., 2025). Chilling stress induces oxidative damage by increasing ROS, which plants mitigate via enhanced antioxidant enzyme activities and osmolyte accumulation like proline and sugars to maintain osmotic balance and membrane stability (Wu et al., 2022). Phytohormones such as abscisic acid play a crucial role in modulating cold stress responses by regulating antioxidant defenses and stress-response genes, thereby improving survival under low temperatures (Feng et al., 2025). Cold stress also impairs photosynthesis by affecting chloroplast structure, Rubisco activity, and the electron transport chain, leading to reduced photosynthetic efficiency. Additionally, plants deploy cryoprotectants like antifreeze proteins and osmolytes to prevent ice formation and cellular dehydration, thus enhancing resilience to freezing conditions (Jahed et al., 2023).

### 2.6 Combined/multiple stresses

Plants exposed to multiple combined stresses, known as multifactorial stress combinations (MFSC), experience a dramatic decline in growth, survival, and overall health even when individual stress levels are low. These complex stress scenarios, increasingly common due to global warming, pollution, and climate change, negatively impact plant physiological, biochemical, and molecular processes, including nutrient assimilation, oxidative balance, and hormone regulation (Zandalinas and Mittler, 2022; Nawaz et al., 2023). Multifactorial stresses also affect crop yield and ecosystem biodiversity by altering microbiomes and metabolic pathways related to energy production and stress tolerance (Priya et al., 2023; Rodríguez-Azorín et al., 2025). The unique nature of plant responses to combined stresses is that tolerance strategies effective against single stresses may fail under combined conditions, highlighting the need for interdisciplinary research to develop climate-resilient crops (Rivero et al., 2021; Jiang Z. et al., 2025). Golaraei et al. (2025) demonstrated that combined stressors such as drought and dust inflicted more severe damage on *Carthamus tinctorius* L. than either stress alone. Drought stress reduced nutrient uptake, soluble sugars, chlorophyll b (by up to 40%), and seed weight per plant, while simultaneously increasing phenolic compounds, carotenoids, and proline. Dust stress similarly decreased phosphorus and soluble sugars, but elevated phenolic content and ascorbic acid levels. To mitigate these effects, a consortium of *Bacillus amyloliquefaciens* and *Bacillus halotolerans* was applied. This PGPB treatment resulted in broad-spectrum improvements, including a 35–64% increase in nitrogen content, enhanced chlorophyll b, and greater plant dry weight and seed yield. In a related study, inoculation with *Azotobacter chroococum* and *Azotobacter vinelandii* alleviated the combined effects of drought and salt stress in eggplant (Kıran and Ellialtioglu, 2023). Furthermore, Staiano et al. (2025) reported that a PGPB consortium comprising *Serratia marcescens*, *Enterobacter cloacae*, and *Bacillus proteolyticus* synergistically enhanced root and shoot growth, chlorophyll and carotenoid content, and antioxidant activity in *Triticum durum* under combined salt and heat stress. Databases like Stress combinations and their interactions in plants (SCIP), facilitate understanding of combined stress responses by integrating phenotypic and omics data to identify key genes and pathways involved in tolerance mechanisms (Priya et al., 2023). Different climate induced stress and their mitigation strategies using plant growth promoting bacteria are summarized in **Table 1**.

**Table 1:** Different stress and their mitigation strategies using plant growth promoting bacteria.

Stress	Microorganisms	PGPB-mediated mode of action	Plant	References
Drought, dust	*Bacillus amyloliquifaciens, Bacillus halotolerance*	Improved nitrogen, chlorophyll content	*Carthamus tinctorius* L	Golaraei et al., 2025
Salt, drought	*Azotobacter chroococum, A. vinelandii*	Increased phytohormone production, remodulation of root system, nutrient absorption	*Solanum mekongena*	Kıran and Ellialtioglu, 2023
Salt, heat	*Serratia marcescens, Enterobacter cloacae*, *Bacillus proteolyticus*	enhanced plant root, shoot growth, chlorophyll, carotenoid content and enhanced antioxidant activity	*Triticum durum*	Staiano et al., 2025
Heat	*Pseudomonas azotoformans*	Improved photosynthetic pigment efficiency, root colonization, growth, and other physiological characteristics.	*Triticum aestivum* L.	Ansari et al., 2021
Salt	*Bacillus* sp., *Pseudomonas* sp.	increase compatible solutes and phenolics	*Glycine max*	Giannelli et al., 2023
Drought	*Pseudomonas palleroniana, Variovorax paradoxus*, *Ochrobactrum anthropi*, *Pseudomonas palleroniana*, *Pseudomonas fluorescens*	Boosted the leaves’ nutritional concentrations and general growth parameters.	*Eleusine coracana*	Chandra et al., 2020
Heat	*Bacillus safensis*	Boost antioxidants and flavonoids, modulating hormone signaling	Beans, maize	Kaleh et al., 2025
Drought	*Pseudomonas* sp. R447, *Trichoderma harzianum* OMG16*Bacillus atrophaeus* Abi03, and	Increase iron uptake, plant hormone balance, antioxidant, increase stress response	*Zea mays* cv. Benedictio	Francioli et al., 2025
Sakine-alkali	*Staphylococcus succinus*	Increased rate of germination, root length and number, and shoot length	*Triticum aestivum*	Xie et al., 2025
Heat, salt,	*Bacillus altitudinis*, *Bacillus cereus*, and *Bacillus velezensis*	Promote morphological enhancement, phosphate solubilization, siderophore production	*Zea mays* L.	Hussain et al., 2025; Kaneriya et al., 2025
Drought	*Bradyrhizobium japonicum, Bradyrhizobium diazoefficien, Bacillus subtilis*, and *Azospirillum brasilense*	Reduced oxidative damage and enhance grain yield, plant growth, and nodulation.	*Glycine max* L.	Moretti et al., 2021
Drought, osmotic	*Chryseobacterium bernardetii*, *Brachybacterium rhamnosum*, *Cytobacillus gottheilii*, *Kocuria palustris*, and *Kitasatospora aureofaciens*	Phytohormone production, siderophore production, increased total soluble sugar like proline	Tea plant	Baruah et al., 2025
Drought	*Azotobacter chroococcum*	Boost soil nutrient availability, growth, and stress tolerance	*Triticum durum*	Silletti et al., 2021
Drought	*Pseudarthrobacter polychromogenes*, and *Paenarthrobacter aurescens*	Enhanced yield, total phenolic compounds, soluble solids content, and irrigation water use efficiency	*Citrullus lanatus*	Yavuz et al., 2025
Saline-alkaline	*Acinetobacter calcoaceticus* DP25, *Enterobacter hormaechei* DP29, and *Staphylococcus epidermidis* DP28	increasing chlorophyll content, antioxidant activity, photosynthetic efficiency, and enzyme activity, modulates electrical conductivity and reduces pH	*Medicago sativa* L.	Han et al., 2025
Drought	*Bacillus*, *Sphingomonas*, *Acinetobacter*, *Leclercia*, *Microbacterium*, and *Brevibacterium*	accumulate secondary metabolites	*Astragalus mongholicus*	Lin et al., 2022
Saline-alkali	*Bacillus* sp., *Aspergillus* sp., and *Penicillium* sp.	Boosted soil nutrient content, reduced soil salinity, and increased soil fertility and enzyme activity	*Oryza sativa*	Guo et al., 2025
Cold	*Pseudomonas* sp. and *Erwinia* sp	producing siderophores, ammonia, and lytic enzymes	*Colobanthus quitensis*	Licciardello et al., 2025
Salinity	*Pseudomonas* sp. SG29+AMF		*Zea mays* L.	Zeng et al., 2025
Salinity, drought	*Pseudomonas fluorescens* RG11+*Funneliformis mosseae, Rhizophagus irregularis*	regulation of expressing stress-responsive genes like VvNCED and VvP5CS	*Vitis vinifera*	Dagher et al., 2025
Drought	*Claroideoglomus claroideum, Burkholderia caledonica*, *Naganishia albida*	increased in photosynthesis, relative water content, nitrogen, phosphorus, and potassium content	Strawberry	Pérez-Moncada et al., 2024
Drought	Pseudomonas putida, Bacillus pumilus, Pseudomonas fluorescens, and Bacillus megaterium	Produced heat shock proteins, bacterial exopolysaccharides, dehydrins, volatile organic molecules, and osmolytes.	Zea mays L.	Kálmán et al., 2024
Drought	*Acaulospora laevis* and *Bacillus subtilus*	Reduced cellular damage, increased root colonization, microbial biomass carbon, and ACC level	*Zea mays* L.	Khan et al., 2024
Salinity	*Pseudomonas* sp.	Increased rates of seed germination, root and shoot lengths, and fresh and dry biomass (biomass from roots and shoots)	*Solanum lycopersicum*	Pandey and Gupta, 2020
Waterlogging	*Pseudomonas* and *Rhizobium*	Improved the fertility and health of the soil, as well as growth and resistance to waterlogging	*Brassica napus* L.	Wasim et al., 2025
Drought	Bacillus cereus	Enhanced the levels of zeatin, gibberellins, IAA, and antioxidant enzyme activity.	Juglans regia	Liu et al., 2023
Drought	*Bacillus velezensis* and *Bacillus amyloliquefaciens*	Increased fruit tree biomass while supporting the plant defense system	*Juglans regia* L.	Lotfi et al., 2022
Salinity	*Bacillus atrophaeus Bacillus megaterium*	Enhanced soil phosphorus (P) concentration, shoot biomass, and shoot P concentration	*Zea mays* L.	Zhu et al., 2023
Drought	*Bacillus megaterium*	Increased auxin production, enhanced antioxidant enzyme, modulate carbon assimilation genes	Sugarcane	Buqori et al., 2025
Salinity	*Bacillus megaterium, B. tequilensis, Pseudomonas putida*	Improved chlorophyll content, reduced electrolyte leakage and water loss, upregulated SOS1 and SOS4 gene	Wheat	Haroon et al., 2022

## 3 PGPB: mechanism of action against climate-induced stress

PGPB reduces the impact of climate-induced stresses like drought, salinity, and heat on plants through various biochemical and physiological mechanisms. Recent studies showed that PGPB helps enhance plant resilience by producing antioxidants, regulating osmotic balance, and modulating hormones. The primary mechanism involves PGPB stimulating the phenylpropanoid pathway, which increases phenolic compounds such as flavonoids (quercetin, catechin) and phenolic acids (caffeic, ferulic) that neutralize ROS generated by oxidative stress. Consequently, phytohormones such as auxins (IAA), gibberellins, and cytokinins are produced, supporting root growth and improving water and nutrient uptake. Additionally, PGPB produce ACC (1-aminocyclopropane-1-carboxylate) deaminase and exopolysaccharides (EPS); the former lowers ethylene levels to reduce stress-induced senescence, while the latter helps retain soil moisture (Jakubowska et al., 2025).

**Figure 1:**
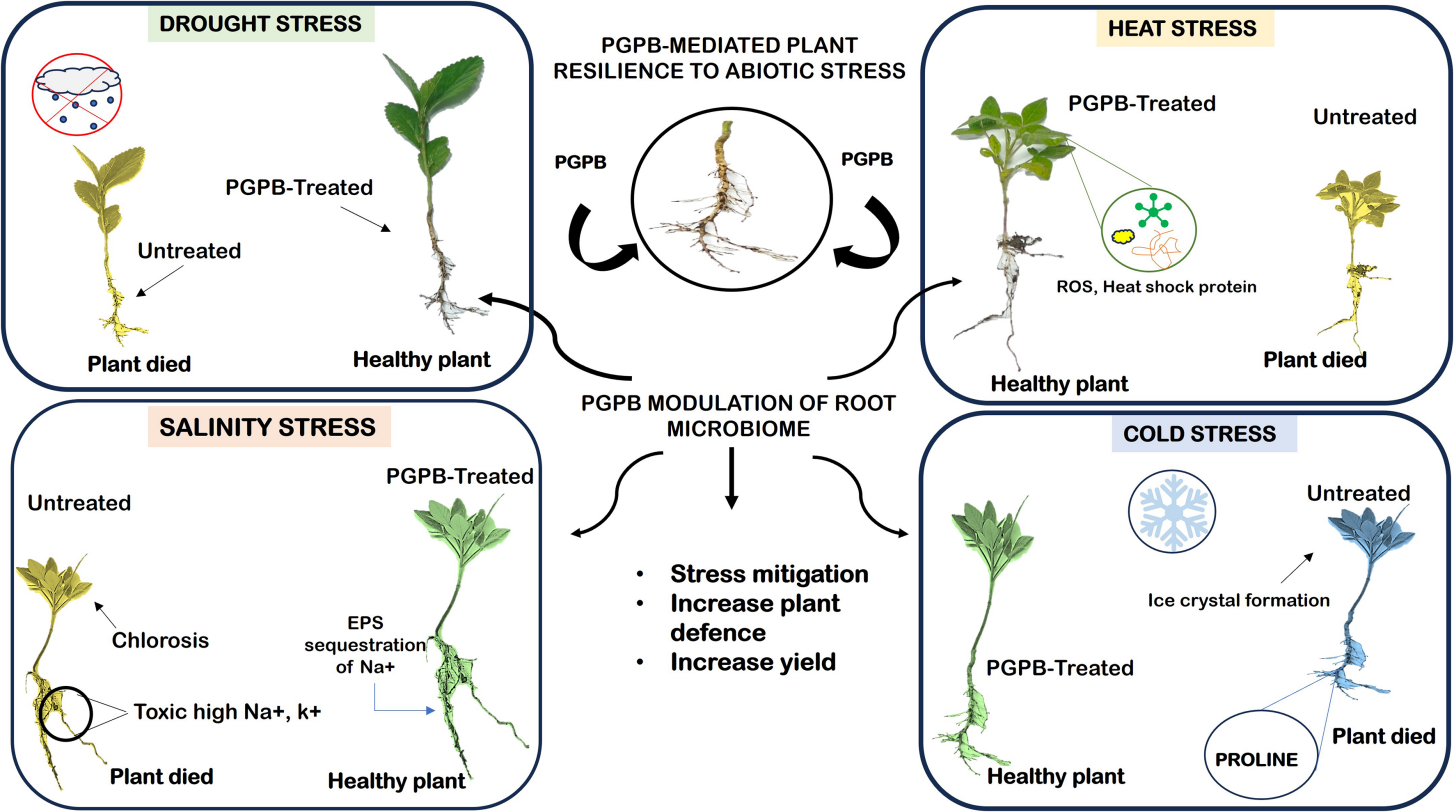
Mechanisms involved in action against climate-induced stress.

In response to drought conditions, PGPB promote osmolytes such as proline and glycine betaine accumulation to balance cellular hydration and turgor, as well as aid in root elongation for water access from deeper parts of the soil (Fadiji et al., 2022). Under salinity conditions, sodium and potassium become rate-limiting factors in regulating ion homeostasis via EPS that bind salts. *Bacillus* and *Pseudomonas* species increase compatible solutes and phenolics, enhancing growth in crops like soybean under high NaCl levels (Giannelli et al., 2023). Auxin-producing PGPB, such as *Bacillus safensis*, reduce oxidative damage (H2O2, MDA) and boost antioxidants and flavonoids, modulating hormone signaling for improved thermotolerance in beans and maize. These mechanisms often synergize, with Induced systemic resistance priming plant defense through jasmonic acid and ethylene signaling pathways, triggering broader stress tolerance responses. Field trials demonstrated 20-60% yield improvements under stress, positioning PGPB as viable, sustainable alternatives to chemical inputs (Kaleh et al., 2025). PGPB also assist crops in coping with cold and flood-related stresses by modulating osmotic balance and metabolic processes. Psychrotolerant Antarctic endophytes such as *Ewingella* and *Pseudomonas* possess genes associated with proline and glycine betaine transport, trehalose metabolism, lipid desaturases, polyamine metabolism, and ROS detoxification (Licciardello et al., 2025). These traits contribute both to bacterial survival and enhanced plant cold tolerance. In tomatoes exposed to 4 °C, these endophytes, together with *Paraburkholderia phytofirmans* PsJN, reduced malondialdehyde accumulation and reprogrammed phenolic metabolism, resulting in elevated levels of 4-hydroxybenzoic acid, salicylic acid, and phenylalanine-derived dipeptides linked to stress responses (Licciardello et al., 2024). PGPB further increased antioxidant enzyme activity, soluble sugars, and proline, while modulating phytohormones to confer cold tolerance. For example, gibberellin-producing *Serratia nematodiphila* enhanced pepper growth under low temperatures by altering GA4, ABA, salicylate, and jasmonate levels (Ali et al., 2022). Research on flooding and waterlogging remains limited; however, several mechanisms have been identified. PGPB commonly induce catalase, ascorbate peroxidase, peroxidase, superoxide dismutase, phenolics, and proline, thereby enabling plants to detoxify ROS generated during hypoxia and subsequent re-oxygenation. Additionally, many PGPB produce osmoprotectants such as proline, trehalose, polyamines, betaines, and exopolysaccharides, which improve osmotic regulation, soil structure, and water movement. These traits are beneficial under flooding, drought, and salinity stress. PGPB also influence root architecture, suberin deposition, and endodermal barrier formation, thereby enhancing mineral balance and resilience to complex stress factors (Grover et al., 2021; Ragland et al., 2024). Mechanisms underlying PGPB-mediated mitigation of climate-induced stress are illustrated in **Figure 1**.

## 4 Synergistic interactions and consortia approaches

Different studies suggested that PGPB often work more effectively in consortia than as individual strains, leading to synergistic effects on plant growth and stress tolerance. Despite the increasing use of bioinoculants from both single strains and consortia, it is important to address strain compatibility to prevent negative interactions such as competition, pathogenicity, and commensalism. For field applications, microbial consortia must be carefully managed and characterized. For example, tests should include responses to external stimuli like pH and temperature, and a rapid, unique plant bioassay and pot experimentation strategy can be employed to determine the best compatibility (Sharma et al., 2023).

### 4.1 PGPB-PGPB consortia interactions

Several studies demonstrated the synergistic effects of microbial consortia that resulted in enhanced root and shoot biomass, nutrient uptake, and flowering more than single strains in wheat, maize, tomato, and other crops (Santoyo et al., 2021; Méndez-Bravo et al., 2023; Utami et al., 2025; Singh et al., 2023). Rhizospheric and endophytic bacteria combinations A8a +B3R7, and SO+B387 all belong to *Bacillus* sp. increased wheat root and dry weight by approximately 34 to 38 percent compared to single inoculations, supporting a synergistic hypothesis where multiple plant growth-promoting traits act cooperatively. Selected PGPR consortia can advance flowering by roughly 30 days and increase plant height by up to 45 percent over controls (Méndez-Bravo et al., 2023). According to Rojas-Solis et al., 2025, microbial consortia such as *Pseudomonas* sp. TL36, *Bacillus* sp. TL80, *Staphylococcus* sp. TL49, and *Gottfrieda* sp. TL52 work synergistically to produce a plethora of plant hormones, siderophores, volatile compounds, and solubilizes phosphate, hence promoting maize growth even under heavy metal stresses like arsenic and mercury. Moreover, microbial consortia such as *Enterobacter ludwigii* EU-BEN-22, *Micrococcus indicus* EU-BRP-6, and *Pseudomonas gessardii* EU-BRK-55 can be used for several horticultural crops grown in hilly regions. They are being used as a combination for their more efficiency compared to single strains (Kaur et al., 2024). A consortium of *Pseudomonas* sp. R447, *Bacillus atrophaeus* Abi03, and *Trichoderma harzianum* OMG16 also synergistically assist plants to alleviate stress responses and adapt to drought conditions in the field (Francioli et al., 2025). Additionally, consortia made up of *Bacillus altitudinis*, *Bacillus cereus*, and *Bacillus velezensis* significantly enhance maize production under heat stress (Hussain et al., 2025), salt stress, and improve maize growth (Kaneriya et al., 2025). For tea plants under drought and osmotic stress, a tolerant consortium including *Chryseobacterium bernardetii*, *Brachybacterium rhamnosum*, *Cytobacillus gottheilii*, *Kocuria palustris*, and *Kitasatospora aureofaciens* has been identified (Baruah et al., 2025). To alleviate saline-alkaline stress, a consortium of *Acinetobacter calcoaceticus* DP25, *Enterobacter hormaechei* DP29, and *Staphylococcus epidermidis* DP28 helps mitigate stress by increasing chlorophyll content, antioxidant activity, photosynthetic efficiency, and enzyme activity. Additionally, it modulates electrical conductivity and reduces pH (Han et al., 2025). Bacterial combinations comprising *Bacillus*, *Sphingomonas*, *Acinetobacter*, *Leclercia*, *Microbacterium*, and *Brevibacterium* are known to accumulate secondary metabolites and promote plant growth under drought stress (Lin et al., 2022). Endophytic microbial consortia from Antarctica’s *Colobanthus quitensis* exhibited remarkable cold tolerance and promote plant growth by producing siderophores, ammonia, and lytic enzymes. They are involved in amino acid metabolism, nitrogen metabolism, zinc transport, potassium transport, and iron transport. Genes responsible for cold stress tolerance, such as those in *Pseudomonas* sp. and *Erwinia* sp., have been identified (Licciardello et al., 2025). These beneficial plant growth-promoting bacteria alleviate biotic stress through the production of antibiotics, hydrolytic enzymes, induced systemic resistance, and the stimulation of antioxidant enzymes (Rana et al., 2025).

### 4.2 PGPB- fungi consortia interactions

PGPB and arbuscular mycorrhizal fungi (AMF) often provide stronger growth promotion and nutrient uptake than either alone in horticultural crops and field species (Santoyo et al., 2021; Emmanuel and Babalola, 2020). Under salinity, maize co-inoculated with AMF and *Pseudomonas* sp. SG29 produced the highest biomass, nutrient uptake, and AMF colonization, outperforming AMF and *Bacillus*, which highlights strain-specific synergy (Zeng et al., 2025). PGPR and AMF inoculation mitigate salt stress in Casuarina and Acacia, as well as drought in maize, by accumulating osmolytes, improving root hydraulics, and enhancing antioxidant activity (Sagar et al., 2021; Khan et al., 2024; Diagne et al., 2020). AMF, particularly *Funneliformis mosseae, Rhizophagus irregularis* combined with bacteria *Pseudomonas fluorescens* RG11, led to a significant increase in salinity and drought tolerance in *Vitis vinifera* (Grapevine). This may be due to the regulation of expressing stress-responsive genes like VvNCED and VvP5CS, which help improved the ROS scavenging, hormonal regulation and osmotic adjustment (Dagher et al., 2025). Moreover, a study done by Pérez-Moncada et al., 2024, revealed that consortia prepared from *Claroideoglomus claroideum, Burkholderia caledonica*, *Naganishia albida* help strawberries tolerate severe drought by reducing lipid peroxidation, and increased in photosynthesis, relative water content, nitrogen, phosphorus, and potassium content. Moreover, their combination redounded on increased fruit yield and overall plant biomass.

### 4.3 PGPB- plant priming agent

Plant priming is a key strategy that prepares the plant defense system to withstand environmental stress. Upon application of a priming agent, the plant enters a primed state characterized by molecular adjustments, with the central mechanism involving modulation of ROS and antioxidant activity. During abiotic stress, ROS levels typically increase; priming induces a controlled ROS spike that functions as a signaling network, stimulating the production of antioxidant enzymes such as superoxide dismutase and catalase (Alum, 2025). In addition, priming reprograms hormonal balance, enabling regulation of stomatal closure and protecting tissues against dehydration. Priming also leaves a chemical imprint on the plant’s DNA structure, for example, by increasing histone acetylation which enhances the expression of stress-responsive genes under adverse climatic conditions. Priming agents may be chemical, physical, or biological (Turhan and Asgher, 2024), with beneficial microbes being the most emphasized. Although priming can be applied flexibly, it is particularly effective during the seed stage, ensuring uniform establishment of priming agents (Rahman et al., 2022).

Priming of plant defenses by plant growth-promoting bacteria involves the activation of jasmonic acid and salicylic acid signaling pathways, which contribute to Induced systemic resistance. This microbial priming enhances phenolic biosynthesis and antioxidant capacity, helping plants tolerate abiotic stress such as salinity, drought, and heavy metals. PGPB strains can activate both salicylic-mediated and Jasmonic acid/ethylene-mediated pathways, protecting against biotic and abiotic stress by priming faster and stronger defense gene expression upon challenge (Galicia-Campos et al., 2024; Maciel-Rodríguez et al., 2025). While exogenous application of salicylic acid or silicon combined with PGPB has not been documented, future research is recommended to explore multi-component formulation and crosstalk between microbial ISR and hormone-mediated priming for improved stress resilience (Darshita et al., 2025). The current evidence highlights that PGPB-mediated ISR is a complex, strain-specific process involving multi-signaling pathways rather than simply mimicking exogenous salicylic acid application. Understanding these interactions could lead to more effective biocontrol consortia and sustainable agriculture practices (Panpatte et al., 2020). Brahim et al. (2026) found that Wheat seeds bio-primed with *Bacillus subtilis* strain MA17, *Bacillus pumilus* strains MA9 and MA19, *Virgibacillus halodenitrificans* strain MA14, showed a high improvement in total length and dry weight of plants compared to unprimed variants under high salt conditions. Mitchener et al. (2025) also demonstrated that there is physiological shifts and shelf-life dynamics of *Brassica napus* seed when bioprimed with *Pseudomonas fluorescens.* Biopriming of *Solanum lycopersicum* with *Pseudomonas* sp. strain VITK-1 and *Burkholderia* sp. strain VITK-3 significantly enhanced overall nutrient solubilization, structural root architecture development, and biochemical stress tolerance pathways (Ragasami et al., 2026). Multispecies assemblies such as PGPB–fungi and PGPB–microalgae consortia are an emerging focus in biopriming, where cross-species interactions are driven by metabolic cross-feeding. For example, microalgae provide oxygen, exopolysaccharides, and carbon sources that protect and nourish bacteria, while the bacteria contribute by fixing nitrogen, synthesizing phytohormones, and solubilizing essential nutrients (Renganathan, 2026). In line with this, da Silva et al. (2024) demonstrated that biopriming soybean with *Parachlorella* sp. and *Bacillus subtilis* in combination with *Trichoderma harzianum* effectively mitigated severe physiological setbacks under saline stress.

## 5 Mechanical bases of synergy

**Figure 2:**
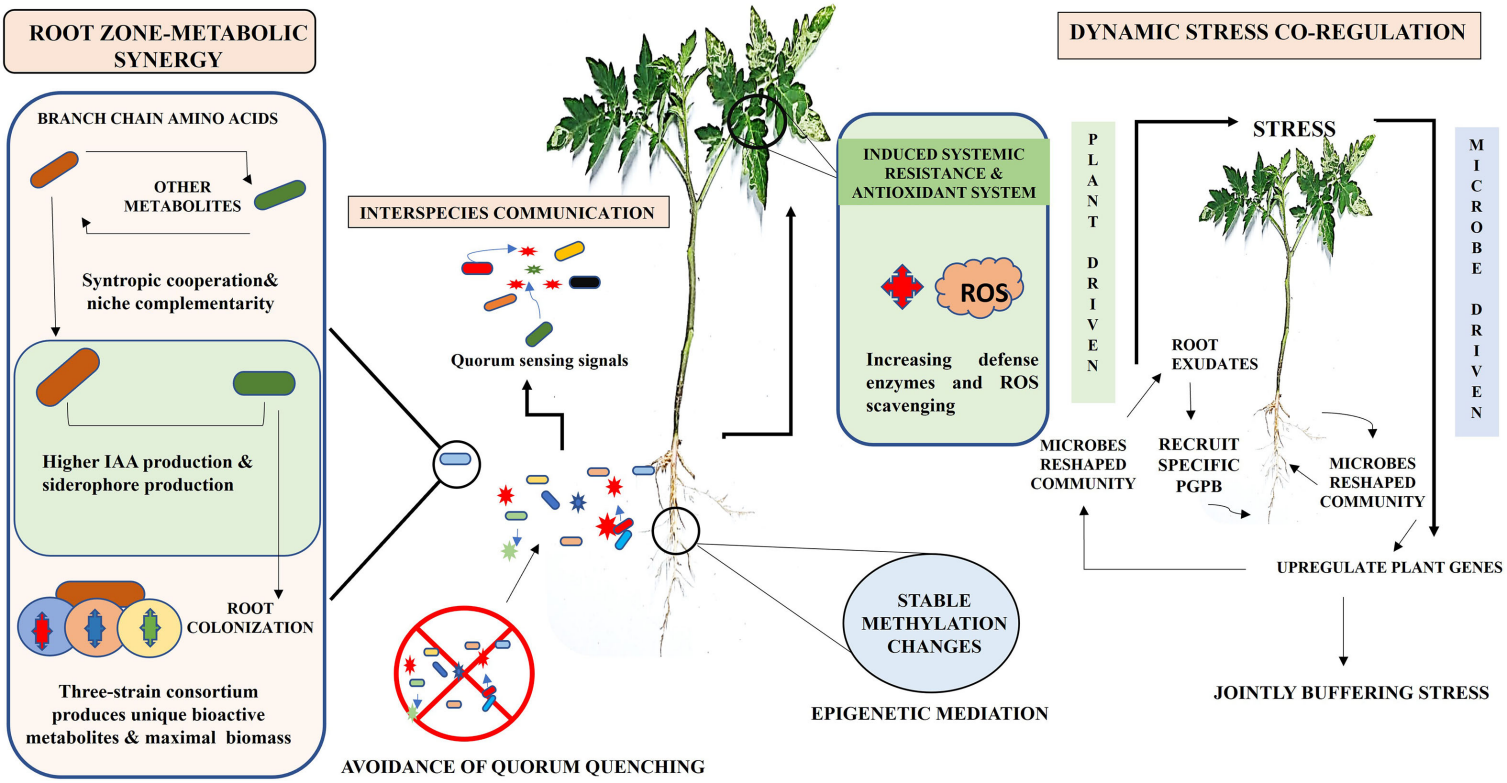
Mechanical bases of synergy among microbes and the plant.

Synergy arises when the interaction between the microbe and plant creates effects greater than the sum of individual strains. Mechanisms are now being understood at biochemical, metabolic, and signaling levels (**Figure 2**).

### 5.1 Metabolic cross-feeding and niche complementarity

*Bacillus velezensis* SQR9 stimulates resident *Pseudomonas stutzeri* through the exchange of branched-chain amino acids and other metabolites; SQR9 supernatant significantly boosts *P. stutzeri* growth, supporting syntropic cooperation and enhanced plant growth and salt tolerance (Sun et al., 2021). Co-cultivation of *Bacillus subtilis* SL-44 and *Enterobacter hormaechei* Wu-15 resulted in a balanced EPS profile and enhanced production of IAA and siderophores, exceeding the levels observed in monocultures and thereby directly strengthening plant growth–promoting traits (Wang et al., 2022). A three-strain consortium of *Azotobacter* sp., *Bacillus megaterium*, and *Pseudomonas* sp. produced unique bioactive metabolites such as N-methyltryptamine, N-6-hydroxy-L-lysine, and sphingolipid intermediates, which was absent in single or two-member cultures and correlated with maximum pigeonpea plant (Srivastava et al., 2023).

### 5.2 Biofilm formation and extracellular matrix cooperation

Biofilm is a complex microbial community that embedded in the extracellular matrix such as, lipids proteins, DNA, and extracellular polymeric substance (EPS), which enables protection, cooperation and resource sharing. For example, *Bacillus* EPS and TasA fibers are essential for interspecies biofilm formation with *P. stutzeri*; deleting these matrix components reduces both partners’ cell numbers and weakens their synergy (Sun et al., 2021). Arnaouteli et al. (2021) also observed that *Bacillus subtilis* mediates aggregation with *Streptococci*, which contributes to interspecies biofilm architecture. The formation of multi-species biofilms is a dynamic process shaped by intricate bacterial interactions that encompasses both the antagonistic and cooperative behavior due to EPS signaling molecules. EPS signaling network yields osmotic stresses, bridging interactions and depletion forces that immobilize the cells, drive phase separation and create spatial niches supporting cooperation (Wang et al., 2022). For instance, in a dual species biofilms formed by *Bacillus* and *Pantoea*, each species contributes matrix elements that confer viscoelastic properties therby protecting *Pantoea* from antimicrobial agents. Multi-species biofilms can form thicker and more structured matrices with altered composition and spatial patterning compared to monocultures, enhancing stress resistance and overall stability (Flemming et al., 2022). Consortia with moderate phylogenetic relatedness form more cooperative swarms and root biofilms, leading to higher IAA, siderophore production, and plant growth than closely related sets (Liu et al., 2025).

### 5.3 Signalling, quorum systems, and plant -level regulation

Synergistic interactions among PGPM often depend on compatible quorum sensing (QS), a chemical signaling mechanism that coordinates microbial traits to enhance plant growth and stress tolerance. Plants, in turn, perceive and reshape these signals, leading to systemic defense activation, antioxidant responses, and even epigenetic reprogramming that can persist beyond the presence of the microbes. QS mediated by N-acyl homoserine lactones (AHLs) and other autoinducers regulates root colonization, hormone production, biofilm formation, and the induction of plant defense (Majdura et al., 2023). The impact of AHLs has been extensively studied across plant systems. The first investigation into plant responses to the complexity of bacterial QS was conducted by Shrestha et al. (2020), who demonstrated that both single AHL molecules and multiple chain molecules significantly promoted the growth of *Arabidopsis thaliana* and induced resistance against *Pseudomonas syringae* pv. tomato (Pst). Through QS, multiple plant growth attributes including biofilm formation and exopolysaccharide (EPS) production are enhanced. For instance, bacteria such as *Pseudomonas aeruginosa*, *P. asiatica*, *P. hunanensis*, and *P. sesami* have been shown to mitigate drought stress, facilitate improved plant growth, and strengthen rhizosphere colonization in maize (Singh and Chauhan, 2022). Avoiding quorum-quenching can disrupt partner traits (Rosier et al., 2020; Singh et al., 2023). Consortia strongly trigger induced systemic resistance and antioxidant systems, enhancing tolerance to pathogens and drought through increased defense enzymes and ROS scavenging (Santoyo et al., 2021; Saleem et al., 2021). Some PGPB can also induce stable DNA methylation changes in roots, reprogramming growth pathways long after bacteria disappear, suggesting epigenetic mediation of lasting synergistic promotion (Chen et al., 2022). QS-guided synthetic consortia of AHL-producing PGPB are suggested as a way to build more reliable, synergistic bioinoculants (Shahni et al., 2025; Hartmann et al., 2021; Negi et al., 2024).

### 5.4 Plant microbe co-regulation under stress

Plants and PGPM act as a co-regulatory unit using root exudates, microbial traits and gene regulation to reduce the stress impacts and sustain growth. Under overgrazing or salinity, plants alter root exudates such as amino acids, sugars, organics acids and secondary metabolites to recruit specific PGPR that fix nitrogen, solubilize phosphate, and produce indole acetic acid, while bacteria reshape the rhizosphere community and upregulate plant genes for hormone production, nutrient transport, and photosynthesis, jointly buffering stress (Jiang H. et al., 2025; Yuan et al., 2025). For example, in *Leymus chinensis*, overgrazing triggers the release of specific root exudates such as L-leucyl-L-alanine and cordycepin, which reshape the rhizosphere microbiome toward communities enriched with PGPB. This process also facilitates colonization by *Phyllobacterium* sp. B68, enhancing chemotaxis and biofilm formation (Yuan et al., 2025). Similarly, the halophyte *Limonium sinense* modifies its root exudate composition under salt stress to recruit *Bacillus flexus*, whose presence contributes to improved stress tolerance (Xiong et al., 2020). Under salinity, plant growth-promoting microbes (PGPM) enhance K+/Na+ homeostasis, osmolyte accumulation, antioxidant capacity, and photosynthetic performance in crops such as rice, wheat, maize, grapevine, and cucumber (Al-Turki et al., 2023; Kumawat et al., 2023; Wang et al., 2025). Moreover, PGPM often induce systemic changes in plant gene expression, upregulating hormone signaling, nutrient and sugar transport, photosynthesis, and ion transport pathways (Ren and Yan, 2026).

## 6 PGPB against climate-induced stress

PGPB modes of action are highly complex, where the full range of mechanisms used for plant growth promotion and stress reduction remains only partly understood. Many studies reported the bacterial mechanisms like nitrogen fixation and phytohormone production, while the molecular responses of many plants to PGPB inoculation are still unknown. In this context, omics-based sciences provide powerful, large-scale, and untargeted approaches to systematically explore these responses and fill the existing knowledge gaps. PGPB inoculation triggers a dynamic and time-dependent transcription factor (TF) response that provides long-term stress tolerance in plants, with WRKY, Ets2 Repressor Factors, and MYB emerging as the main TF families across inoculated plant systems. These TFs act as key regulators that control the expression of defense genes, growth-promoting genes, and secondary metabolite biosynthesis under both biotic and abiotic stress conditions. After PGPB priming, ERF and WRKY TFs are most consistently activated in pathogen-challenged plants, while ERF and DREB are the main TFs expressed under various abiotic stress conditions, highlighting ERF’s dual regulatory role across different stress types. Despite these advancements, important research gaps persist, especially regarding the mechanisms of temporal TF regulation after priming and the characterization of TF responses in a wider range of biotic and abiotic stress scenarios, emphasizing the need for more comprehensive studies to fully understand the PGPB-mediated priming network (Kaleh et al., 2025). From the proteomics studies, it has been shown that PGPB boosts the expression of ROS-scavenging proteins, heat shock proteins (HSPs), and proteasomes in stressed plants, aiding protein processing and oxidative stress mitigation. For instance, HSP70 upregulation occurs post-PGPB under heavy metal stress, overlapping with drought responses. Nevertheless, proteome analysis of plant responses to PGPB remains a considerably underexplored field, and substantial further investigation is required before robust and generalizable conclusions can be drawn (Rodríguez-Vázquez and Mesa-Marin, 2023). Additionally, metagenomic studies have shown that inoculation with PGPB, including strains such as *Bacillus* and *Enterobacter*, significantly alters the rhizosphere microbiome’s composition and function under stress conditions. Under combined stresses such as microplastic-cadmium contamination, PGPB enriches key groups like Pseudomonadota, Actinomycetota, and Acidobacteriota, while increasing overall microbial diversity and functions such as glycosaminoglycan biosynthesis. These community changes lead to enhanced ecosystem functions, including phosphorus and nitrogen solubilization, pathogen suppression through competitive exclusion, and notable plant growth increases, with dry weight rising by up to 42% in Pennisetum. Similarly, under climate-related abiotic stresses like drought and salinity, PGPB inoculation reorganizes rhizosphere communities to favor osmolyte-producing and nitrogen-fixing microbes, which collectively boost plant tolerance and resilience (Zhao et al., 2025).

## 7 Lab to field: major constrains

Lab-proven plant growth-promoting bacteria often underperform in the field because soil conditions and native microbiota strongly limit their survival, colonization, and expression of PGP traits; better selection and formulation strategies can narrow this gap (Hossain et al., 2023). PGPB performance is linked to soil pH, temperature, moisture, and nutrient levels. For example, neutral pH, moderate temperature, and sufficient moisture encourage colonization, while extreme conditions sharply reduce effectiveness (Lopes et al., 2021). Soil chemistry, including nutrient content, contamination levels, and fertility, also influences colonization; high-nutrient soils can decrease root colonization as microbes migrate to richer microsites. pH significantly shapes community composition and the home-field advantage of native microbes; isolates that are highly efficient *in vitro*, for instance, can be ineffective in native soil because they never become dominant populations (Feng et al., 2023). Another important challenge is that introduced PGPB frequently exhibit reduced competitiveness against well-adapted native microbial communities, resulting in persistently low population densities that constrain their functional contributions. Additionally, native microbiota structure and diversity are crucial for soil health and can buffer or reduce inoculant effects; many studies showed a large plant response but relatively minor community shifts, or vice versa. Consortia made from native strains can colonize more effectively and promote growth better than commercial PGPR in low-fertility soils, while commercial strains may struggle to establish and could increase niche competition (Kulkova et al., 2023; Dobrzyński et al., 2025; Jiang et al., 2023). Therefore, to effectively translate laboratory findings into reliable field performance, it is essential to adopt more robust and context-specific strategies. Critical environmental variables such as soil pH, temperature, moisture content, and the composition of native microbial communities must be systematically considered. Furthermore, the development of stable formulations, the use of locally adapted microbial strains, and the implementation of phased field validation trials are pivotal. Collectively, these approaches can substantially enhance the consistency and efficacy of outcomes when transitioning from controlled experimental conditions to heterogeneous field environments (d’Amelio et al., 2025).

## 8 Formulation and application strategies

Research on plant growth-promoting bacteria has progressed from basic peat-based rhizobia inoculants like Nitragin to advanced encapsulated consortia and versatile formulations that enhance shelf life and offer flexible application methods (Santos and Reis, 2025). Certain formulation strategies and carrier materials are discussed below.

### 8.1 Classic solid carrier

Peat is the standard carrier for rhizobia and many PGPB. It is well characterized and widely used due to their high organic matter, physical protection nutrient supply and their ability to support bacterial survival under water and heat stress. However, peat is slowly renewable resources with limited availability in some regions, prompting research into alternatives including compost, coir dust/coco peat, sawdust, charcoal, sand, and perlite. Studies have shown that cork compost and perlite can maintain rhizobia survival as well, or better than peat, while municipal solid waste compost also supports high rhizobia population comparable to peat (Akuru et al., 2024). The use of peat as a primary medium and carrier for PGPB has been increasingly scrutinized. Recent studies highlight that peat poses inherent chemical and environmental phytotoxicity risks, eliciting highly species-specific responses in target crops due to its high content of phenolics, tannins, and humic–fulvic complexes. Moreover, the elevated acidity of peat enhances the bioavailability of potentially toxic heavy metals, which can inhibit cell division. Therefore, careful selection of plant species is essential when employing peat as a substrate for delivering microbial formulations (Alsafran et al., 2022; Cui et al., 2024).

### 8.2 Talc or other mineral carriers

Common carrier materials such as talc, sand, vermiculite, and charcoal are widely used for seed and soil inoculation due to their low cost and ease of formulation. However, the choice of carrier can significantly influence microbial establishment in the rhizosphere. For instance, in the case of non-sporulating *Pseudomonas* spp., peat-based formulations generally support higher rhizospheric colonization compared to talc-based carriers, indicating that talc may be less effective in sustaining microbial viability and activity after application (Novinscak and Filion, 2020).

### 8.3 Alginate beads

Sodium alginate 1-3% with 1-2% CaCl2 is widely used for bioencapsulation. It is known for its ability to protect cells from desiccation and UV, enable controlled release, and maintain viability and shelf life for 2–4 years in rhizobia and other PGPB. Encapsulated formulations are especially valuable for non-sporulating gram negative and gram negatives consortia (Fanai et al., 2024; Etesami, 2025).

### 8.4 Liquid formulations

Whole-cell cultures are often supplemented with stabilizing agents such as oils and polymers including polyvinylpyrrolidone (PVP), carboxymethyl cellulose (CMC), natural gums, alginate, and glycerol to enhance microbial survival during storage and improve performance following application. These additives also facilitate microbial adhesion to seeds and soil particles, thereby promoting effective colonization. Such formulations are widely employed for beneficial microorganisms, including *Rhizobium, Azospirillum*, and *Pseudomonas* (Gómez-Godínez et al., 2023). Notably, alginate encapsulation and other polymer-based delivery systems are increasingly recognized as next-generation technologies for both single-strain inoculants and synthetic microbial consortia (SynComs), as they enhance stress tolerance and enable more controlled and targeted delivery under field conditions (Ayuba et al., 2026).

## 9 Application of bio-formulants

### 9.1 Seed treatment or biopriming

Biopriming of seed is a pre-sowing treatment where seeds are hydrated with the live inoculum, followed by drying, which allows bacteria to adhere to and enter the seeds, form a protective layer on the seeds, favoring colonization, thus helping them acclimate. This process enhances the germination rate and strengthens stress tolerance. Seed treatment is often preferred to maximize early root colonization and growth stimulation. Lyophilized seed coating can dramatically improve survival rate and seedling emergence, compared to simple carrier-based coating. Seed biopriming ensures uniform emergence, stress tolerance, and allows acclimatization and adherence of PGPR in the seeds (Orozco-Mosqueda et al., 2021; Etesami, 2025). Biopriming with formulants raises tolerance to salinity and other stresses by enhancing osmolyte metabolism, antioxidant system, nutrient uptake and root exploration. Moreover, it activates antioxidant systems, osmotic adjustments and stress responsive genes/MAPK pathways that can persist into later growth stages (Jatana et al., 2024). Microbial biopriming reduces seed and soil borne diseases through competition, antibiosis, cell wall degradation induced systemic resistance and improve seed defense metabolism (Fiodor et al., 2023; Srivastava et al., 2023). Seed biopriming is often preferred for their efficiency, low doses whose single application resulted in strong early root colonization and rhizosphere establishment even under stress, improving yield potential and soil health (Makhaye et al., 2021).

### 9.2 Soil application or drenching

This application approach facilitates broad distribution of inoculants throughout the root zone; however, it typically requires relatively large inoculum volumes and is prone to losses through leaching and soil movement. In addition, scalability can be a limiting factor under field conditions. The use of carrier-based or encapsulated formulations (such as alginate beads applied directly to the rhizosphere) can mitigate these losses by enabling controlled and sustained release of microbial cells. Alternatively, liquid and peat-based formulations may be delivered via in-furrow application, banding, or soil drenching. These methods are particularly advantageous in situations where seed treatment is constrained or incompatible with pesticide use (O'Callaghan et al., 2022; Duarte et al., 2024). For instance, Nicotra et al. (2025) showed soil drenching with PGPR improved tomato growth under drought stress by altering rhizosphere communities in a strain-specific way, highlighting the potential of microbial consortia and individual PGPM as sustainable tools to improve plant resilience to abiotic stresses. A microbial consortium made up of *Bactterium* 76A and *Trichoderma* T22 increase tomato yield by approximately 48% (Cirillo et al., 2023). Soil application with *Bacilus* -*Pseudomonas*-*Trichoderma* consortium enhanced maize growth, iron uptake and drought adaptation in field conditions (Francioli et al., 2025). Potato inoculated with arbuscular mycorrhizal (AM) fungi and *Bacillus* in combination with silicate soil exhibited higher biomass and tuber yield under drought conditions (Mamun et al., 2024). Similarly, a field experiment conducted by Ashwin et al. (2023) demonstrated that the combined application of *Bradyrhizobium liaoningense* and *Ambispora leptoticha* improved the overall performance of the drought-susceptible soybean cultivar MAUS-2 when exposed to drought stress.

### 9.3 Foliar application (phyllosphere)

Foliar applications are targeted; therefore, they require less inoculum compared to the drenching method. However, upon field application, microbial cells are exposed to environmental stresses such as desiccation, ultraviolet (UV) radiation, and fluctuating weather conditions, all of which can markedly reduce their viability. Consequently, these stresses often lead to inconsistent field performance unless protective and optimized formulations are employed to enhance microbial survival and functional stability (Orozco-Mosqueda et al., 2021).

Comparative evaluations of PGPB inoculation strategies under heat stress indicate that alginate-encapsulated formulations and soil drenching are among the most effective and scalable approaches. Alginate beads provide enhanced protection to microbial cells and enable controlled release, improving survival under thermal stress. Seed coating, in contrast, localizes bacterial populations at the root surface, facilitating early colonization, whereas soil drenching promotes broader distribution within the rhizosphere but may involve increased risks of phytotoxic effects depending on formulation and dosage. Alginate encapsulation notably improves bacterial survival under desiccation and UV exposure, maintaining high viability over long term storage while supporting early plant growth and nodulation (Balla et al., 2025). Non-sporulating gram negative PGPB are particularly challenging to stabilize, requiring advanced technique such as freeze drying, liquid stabilizers, and encapsulation; novel matrices like pickering emulsions enhanced survival even when co-delivering pesticides, enabling combined biological and chemical applications (De-Bashan et al., 2024). Challenges in microbial formulations includes shelf life and viability maintenance, with compatibility testing an ecological fit being crucial for consortia rather than simply mixing high performing strains (Hossain et al., 2023; Orozco-Mosqueda et al., 2021). Low-cost carrier and formulation also impact shelf life and efficacy, with some agricultural waste-based carrier showing promise but varying stability depending on sterilization and environmental conditions (Mwafulirwa and Kanyada, 2023). Overall, alginate encapsulation emerges as a robust and effective strategy for preserving the viability of PGPB during both storage and field application. This approach supports the development of tailored inoculation strategies that can be optimized according to crop developmental stage and prevailing environmental conditions, thereby enhancing the consistency and efficacy of microbial performance in agricultural systems (Balla et al., 2025).

## 10 Future perspective

### 10.1 SynCom strategy for climate-smart agriculture

Synthetic microbial communities, commonly known as SynComs, are assembled consortia that carry functional traits capable of aiding nutrient cycling, hormone modulation, and plant defense. In one study, a five-strain tomato SynCom mitigated polyethylene glycol–induced drought damage *in vitro* and improved water stress responses, as well as rhizosphere diversity, under 40% reduced irrigation in greenhouse conditions, showing both strain-specific and community-level effects (Nicotra et al., 2025). Additionally, a five-strain SynCom derived from *Indigofera argentea* conferred salt stress resilience to tomato in non-sterile soil, which was associated with altered expression of salt stress–related genes and ion accumulation. A five-strain Gram-negative SynCom with phosphate-solubilizing, auxin-producing, and nitrogen-fixing traits modulated the physiology and metabolome of *Salvia officinalis*. Under both optimal and reduced irrigation, inoculation with this SynCom induced drought-like physiological adjustments and altered metabolites such as histamine and alpha-ketoglutarate, suggesting that these communities do not simply promote growth but also precondition the plant (Jaoudé et al., 2025). Overall, these SynCom studies propose a new perspective on consortia development, marking a shift from empirical approaches to rational, trait- and model-informed community design, with an emphasis on minimal yet robust consortia for stress-resilient, climate-smart agriculture.

### 10.2 CRISPR-engineered PGPB

CRISPR technology is increasingly used to engineer PGPB and crop plants to boost stress tolerance, especially against abiotic stresses like salinity, drought, and heat. CRISPR-Cas systems enable precise genetic modifications in both plants and their associated microbiomes, improving nutrient uptake, stress resilience, and pathogen resistance without introducing foreign DNA. This may ease regulatory concerns and facilitate commercialization (Kumawat et al., 2023; Rahman et al., 2022; Wang et al., 2025). AI and machine learning tools are being integrated with genomic and phenomic data to predict microbiome-crop interactions and accelerate the breeding of climate-resilient crops through high-throughput phenotyping and big data analytics (Razzaq et al., 2021). Microbial inoculants such as PGPRs and arbuscular mycorrhizal fungi work together with biotechnological advances to improve soil health, immunity, and yield under environmental stresses, supporting climate-smart agriculture practices (Srivastava et al., 2025). Challenges remain in strain specificity, field application scalability, regulatory frameworks, and farmer acceptance; however, the combination of CRISPR-engineered microbes and crops holds strong potential for sustainable bioeconomy development by reducing agrochemical inputs while increasing productivity. Overall, integrating CRISPR genome editing with AI-driven microbiome management represents an exciting frontier for advancing climate-smart agriculture and commercializing crop-microbiome systems (Al-Zubaie, 2025).

## 11 Conclusion

Climate change is intensifying extreme weather events, including rising temperatures, prolonged droughts, irregular rainfall, soil salinization, flooding, and heatwaves, all of which disrupt plant systems, reduce yields, and threaten global food security. These stresses often occur simultaneously, creating multifactorial conditions that are more severe than any single stressor and pose significant challenges to agricultural productivity. Traditional practices reliant on chemical fertilizers and pesticides not only fail to address these issues but can exacerbate climate-induced stress, underscoring the need for eco-friendly and resilient alternatives. PGPB offer a sustainable strategy to enhance resilience through bioactive metabolites that regulate nutrient cycling, antioxidant defense, and stress-responsive pathways. Among microbial approaches, consortia of plant growth-promoting microbes (PGPM) are particularly effective, as synergistic assemblies generally outperform single strains due to their broader functional capacity and adaptability. Such consortia mitigate drought, salinity, heat, and osmotic imbalance while promoting plant growth. For instance, drought-tolerant bacteria improve water-use efficiency, while salt-tolerant strains help balance ions and reduce sodium toxicity. Cooperative microbial functioning also boosts ecological fitness, ensuring survival under environmental fluctuations and enhancing adaptability in field conditions. Stress alleviation mechanisms involve diverse and interconnected pathways. The phenylpropanoid biosynthetic pathway, for example, increases phenolic compounds, flavonoids, and lignin, which act as antioxidants to scavenge ROS and protect plant cells from oxidative damage. Beneficial microbes also produce phytohormones such as IAA, gibberellins, cytokinins, and abscisic acid, influencing growth and stress signaling. Another crucial mechanism is the production of ACC deaminase, which reduces stress-induced ethylene levels. Additionally, microbial exopolysaccharides enhance soil structure, improve moisture retention, protect microbial cells from desiccation, and aid osmotic adjustment, thereby supporting plant survival under drought conditions. Despite strong laboratory evidence, field performance remains inconsistent due to environmental variability that affects microbial survival, colonization, and plant–microbe interactions. Introduced inoculants must also compete with complex native soil microbiota, which can suppress or alter their activity. To address these challenges, recent advances in formulation strategies, synthetic microbial communities (SynComs), CRISPR-based engineering, and AI-driven microbiome management offer promising directions for climate-smart agriculture. Nevertheless, critical research gaps persist. Microbial synergy depends strongly on strain combinations, host specificity, and environmental context, with interactions ranging from beneficial to neutral or even antagonistic. Many consortia that perform well under controlled conditions fail to establish stable relationships in open fields. Furthermore, the long-term stability, ecological effectiveness, and performance of microbial consortia across multiple cropping seasons remain poorly understood. There is a clear need for systematic, long-term field validation and deeper investigation into interactions with native soil microbiota to enable the development of robust, reliable, and scalable bioformulations. Such efforts are essential to support sustainable crop production and safeguard food security in the face of changing climates.
